# The Pursuit of Chronically Reliable Neural Interfaces: A Materials Perspective

**DOI:** 10.3389/fnins.2016.00599

**Published:** 2016-12-27

**Authors:** Liang Guo

**Affiliations:** ^1^Department of Electrical and Computer Engineering, The Ohio State UniversityColumbus, OH, USA; ^2^Department of Neuroscience, The Ohio State UniversityColumbus, OH, USA

**Keywords:** stretchable electronics, conducting polymer, neural interface, microelectrode array, biomimicry

## Abstract

Brain–computer interfaces represent one of the most astonishing technologies in our era. However, the grand challenge of chronic instability and limited throughput of the electrode–tissue interface has significantly hindered the further development and ultimate deployment of such exciting technologies. A multidisciplinary research workforce has been called upon to respond to this engineering need. In this paper, I briefly review this multidisciplinary pursuit of chronically reliable neural interfaces from a materials perspective by analyzing the problem, abstracting the engineering principles, and summarizing the corresponding engineering strategies. I further draw my future perspectives by extending the proposed engineering principles.

## Introduction

The fascination of mind-controlled machines often seen in science fiction movies has reflected a recent passionate pursuit of such neurotechnologies by an integrative community of scientists, engineers, and physicians. Collectively, this broad field is named *Neural Prosthetics*, yet its borders are being constantly shaped by fast evolving new technological advances. Although the science fiction has come closer to reality with the demonstration of possibility in humans (Hochberg et al., 2012; Collinger et al., 2013; Bouton et al., 2016), a feasible system for long-term daily use is still far out of reach, primarily due to the discovery of fibrotic encapsulation developing around the implanted neural interface over a short time window of a few months, which physically screens the electrical sensors from accessing to the target neurons (Rousche and Normann, 1998; Jorfi et al., 2015). This hassle has somehow resulted in a brief cooling down of the initial intense enthusiasm in the scientific community and the general public and a halt in the rush to commercialization of such heavily invasive neurotechnologies. Correspondingly, the field's focus has been steered toward scrutinizing this problem of chronic instability of neural interfacing, with an ambition to address it in this decade.

More recently, stimulated by advocations and funding supports on brain-related research across the US, Europe, and Asia, the once electrical-engineering concentrated field starts to bloom, attracting an ever large, and diverse research workforce who brings in invaluable multidisciplinary perspectives and expertise in reforming the field. On the one hand, increasingly more signal channels are being integrated in neural implants to boost the bandwidth of the acquired data in large-scale recording (Berényi et al., 2014; Ruther and Paul, 2015; Shobe et al., 2015), reflecting strong contributions from the traditional community. On the other hand, as the mechanisms of short-term and long-term tissue responses to neural implants are being unveiled (Biran et al., 2005; Polikov et al., 2005; Grill et al., 2009; Kotov et al., 2009; Marin and Fernandez, 2010; Potter et al., 2012; Kozai et al., 2012a, 2014, 2015a,b; Jorfi et al., 2015) materials science and tissue engineering have shown increasing importance as essential add-ons to the research enterprise in developing advanced neural interfaces (Aravamudhan and Bellamkonda, 2011).

In this paper, I will briefly review this multidisciplinary pursuit of chronically reliable neural interfaces from a materials perspective by analyzing the problem, abstracting the engineering principles, and summarizing the corresponding engineering strategies.

## The grand challenge and the ideal neural interface

The *grand challenge* to chronically reliable neural interfacing is *instability* and *limited throughput* of the electrode–tissue interface, which is universal to all current implanted neural interfaces. Ideally, a neural prosthesis should function as if it is part of our native body, in terms of both cognitive control and perception. Correspondingly, the *ideal neural interface* needs to be physiologically integrated to the target neural tissue at the tissue and cellular levels with high fidelity over the life span of the host. This high-fidelity physiological integration requires long-term stability in physical integration and long-term stability and sufficient bandwidth in functional integration. I will elucidate these two aspects in details in the following sections.

## Physical integration

The engineering challenge to stable physical integration stems from different classes of materials involved at the electrode–tissue interface, with the implanted abiologic materials subject to immune regulation.

### Engineering principles

In order to address the stability issue to physical integration of current neural interfaces, two alternative engineering principles are being explored aiming at making the neural implant either *insensible* or *indistinguishable* to the host tissue environment.

#### Insensibility

**Figure 1:**
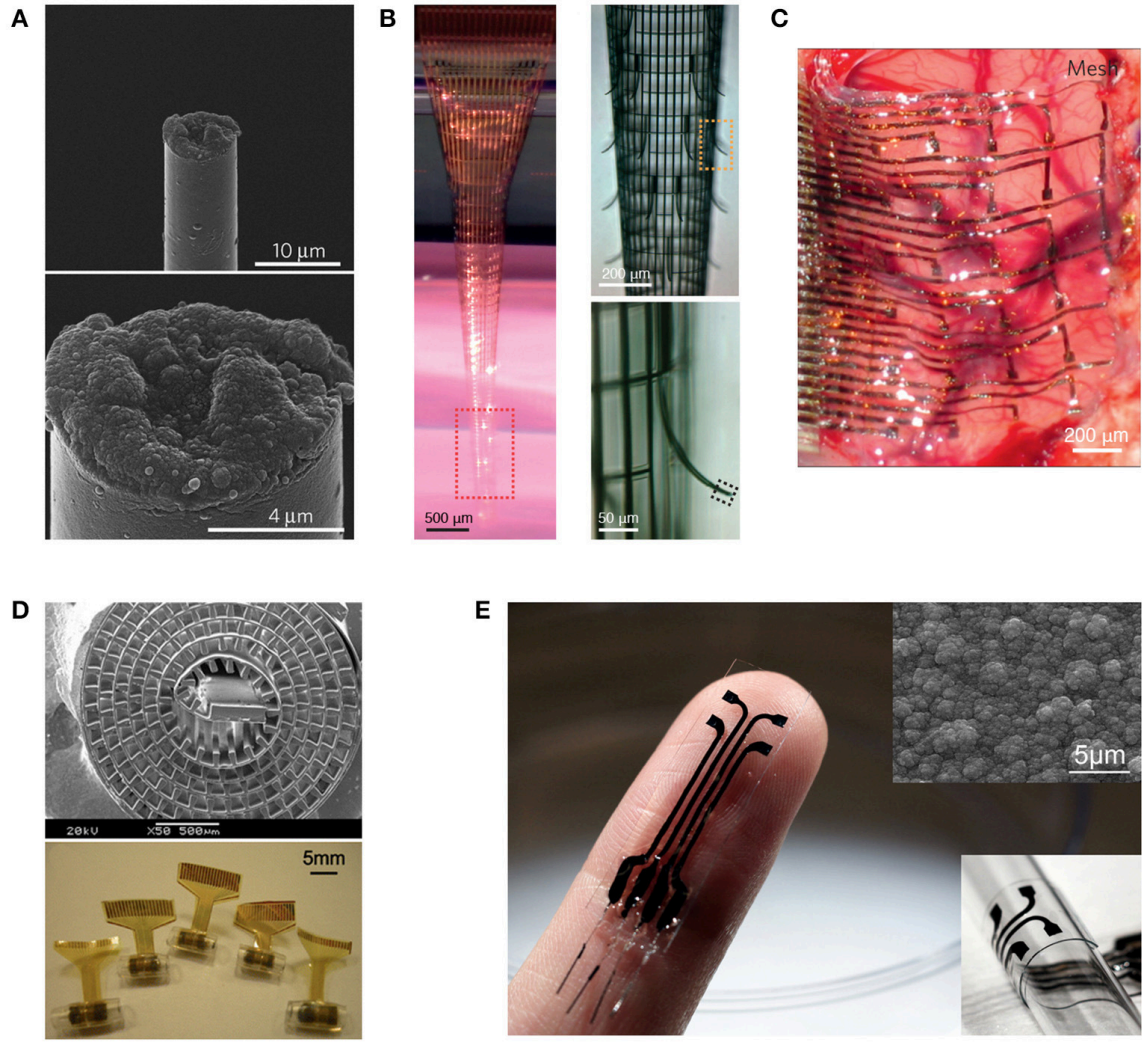
**Representative neural interfaces reflecting recent technological evolution. (A)** Microthread electrode (Kozai et al., 2012b), **(B)** Macroporous nanoelectrode array (Xie et al., 2015), **(C)** Compliant electrode array for electrocorticography (Kim et al., 2010), **(D)** Regenerative neural electrode array for peripheral nerve interfacing (Lacour et al., 2010), and **(E)** Stretchable polymeric multielectrode array for conformal neural interfacing (Guo et al., 2014). Copyright permissions were obtained from the respective publishers.

For the insensibility principle, if the host tissue cannot perceive the existence of the implant, there should be no immune response. Although it is impossible to completely make an implant physically insensible, reduction of its footprint down to the micro/nanoscale has proven to dramatically improve the chronic stability of the neural interface (Kozai et al., 2012b; Xie et al., 2015). This engineering principle is straightforward to implement and often applied together with the indistinguishability principle (Figure 1A; Kozai et al., 2012b). Its recent manifestation in nanoelectronic neural interfaces shows great promise (Figure 1B; Xie et al., 2015).

#### Indistinguishability

For the indistinguishability principle, alternatively, if the implant materials can be camouflaged so that the cells, particularly the immune cells, would discern minimal differences between the abiologic materials and their native environment, there should be minimal immune response, either. In line with this engineering principle, the ideal neural implant should be able to surpass the immune surveillance by mimicking the host tissue environment both physically and biochemically, thus making itself indistinguishable. In the next section, I will describe two primary biomimicry engineering strategies under this principle, which have been intensively pursued in the field to improve the physical integration of neural interfaces, as related to our own work in the past decade.

### Biomimicry strategies for stable physical integration

Cells reside in their extracellular matrix (ECM) with which they interact physically and biochemically. If the implant materials are made indistinguishable from the ECM, both the immune cells and the target neurons will be fooled, and as a result, neither induction of fibrosis nor expulsion of the neurons should happen. Based on this principle, two representative engineering strategies have been explored from the mechanical and biochemical aspects respectively for biomimicry.

#### Mechanical mimicry: from rigid to soft interfaces

The field of neural interfaces has passed a few prominent milestones. The transition from single-needle electrode to multiwire electrodes dramatically increased the information that could be extracted from the nervous system. The introduction of CMOS microfabrication technologies to the fabrication of multichannel neural microelectrode arrays offered unprecedented spatial accuracy and reproducibility to neural interfacing. The renowned Utah Arrays and Michigan Probes are the classic examples of such implantable interfaces. Many excellent reviews are available on these rigid neural interfaces (Cheung, 2007; Heer and Hierlemann, 2007; Ghane-Motlagh and Sawan, 2013; Patil and Thakor, 2016). It is only within the past decade or so that the scientific community started to steer toward making soft versions of these devices, as one prominent approach to reduce the neuro-inflammation response and improve the chronic performance (Grill et al., 2009; Kotov et al., 2009; Nguyen et al., 2014). This is an essential stage for the development of long-term reliable neural prostheses, as contemporary technologies and regulations have advanced sufficient to permit chronic studies. As a result, numerous flexible and stretchable microelectrode arrays have emerged, first as surface interfaces (Figure 1C) and later coming up with intracortical probes when a range of insertion mechanisms were developed (Patil and Thakor, 2016). More recently, the concept of regenerative neural interfaces is being revived, combining state-of-the-art stretchable electronics with tissue engineering approaches for a better-integrated electrode-tissue interface (Figure 1D; Lacour et al., 2010; Clements et al., 2013; Musick et al., 2015; Srinivasan et al., 2015; Thompson et al., 2016). All these moves manifest the pursuit of a biomimicry strategy from the mechanical aspect under the indistinguishability principle. By making neural implants of soft materials that have mechanical moduli closer to those of the host soft tissues, it is intended to minimize the mechanical stiffness mismatch between the implant and surrounding soft tissues, so that the cells feel mechanically more similar to their native environment at the interface and react less wildly, resulting in reduction of both short-term inflammation and long-term fibrotic encapsulation (Kotov et al., 2009; Nguyen et al., 2014).

#### Biochemical mimicry: from metal to polymer conductors

Major research efforts have been devoted to biomaterial surface-modification of existing rigid neural interfaces to mimic the ECM, so that the cells feel biochemically similar to their native environment at the interface (Kolarcik et al., 2012; Martin, 2015). Furthermore, if the implant materials can release anti-inflammatory drugs and neurotrophins, while suppressing implantation-induced trauma, the implant can induce target neurons or their axons to grow to the electrode site for an intimate integration. Although the non-conducting parts of a neural implant can be coated with appropriate dielectric polymers to facilitate bio-functionalization, direct bio-functionalization on metal electrodes while preserving their electrical conductivity is difficult. Conducting Polymers (CPs) are unique in accommodating electrical functionality with ECM biomimicry through bio-functionalization and have conventionally been used in electroactive tissue scaffolds (Hardy et al., 2013). They are also capable of releasing anti-inflammatory drugs and neurotrophins in an electrically controlled manner (Svirskis et al., 2010). Therefore, the application of CPs as neural electrode coatings to offer a localized conducive microenvironment for intimate neuron–electrode integration has been extensive investigated in the past decade (Guimard et al., 2007; Green et al., 2008; Ravichandran et al., 2010; Yi and Abidian, 2015).

However, one of the major drawbacks of CP electrode coatings is the delamination issue, making the coating less durable, and the electrical property of the electrode less stable. Moreover, conventional CPs are brittle, making it difficult for soft neural electrodes to incorporate CP coatings. Leveraging our recent invention of a mechanically strong polypyrrole composite (Ma et al., 2013), we have developed a stretchable polymeric multielectrode array using the polypyrrole composite as the sole conductor for both the electrode and interconnects (Figure 1E; Guo et al., 2014). This is the first neural interface that can offer the benefits of CP electrodes in a demanding stretchable format, as well as the first stretchable neural interface that uses a CP film as the sole conductor. This opens up the opportunities for convenient bio-functionalization of the electrodes using well-established physical and chemical methods (Guimard et al., 2007; Green et al., 2008; Ravichandran et al., 2010), which can be combined with the mechanical biomimicry strategy to further embody the indistinguishability principle.

## Functional integration

The engineering challenge to stable and efficient functional integration arises as a result of the different communication mechanisms employed at the electrode-tissue interface, with ionic/biochemical signals and information coding mechanisms yet not fully understood on the biologic side in contrast to the electronic signals and the artificial coding mechanism on the abiologic side (Schalk, 2008).

### Engineering principle

The principle for functional integration is *seamlessness* that requires both long-term stability and sufficient bandwidth for reliable and efficient communication.

### Biomimicry strategy for stable functional integration

In order to achieve long-term stable electrical signal transduction at the electrode-tissue interface, it is necessary to mimic the biologic ionic charge transfer mechanism, so that sufficient charge can be transported across the interface without disturbing the normal physiological microenvironment through induction of irreversible faradaic reactions, particularly during neural stimulation. To this end, CPs, particularly poly(3,4-ethylenedioxythiophene) (PEDOT), are very attractive in meeting this requirement as the electrode material (Martin, 2015). They conduct electrical current in their conjugated backbone via electrons and across their surface–electrolyte interface via reversible doping ion exchange (Guo, 2016), making them an ideal electronic–ionic charge transducer between the electronics and the neural tissue. Combining with their other properties including bio-functionalization and electrically controlled drug release, CP electrodes offer opportunities for implementing the biomimicry engineering strategies in both the physical and functional integration aspects. No wonder that the research interests on CP neural electrodes are intense during recent years (Guimard et al., 2007; Green et al., 2008; Ravichandran et al., 2010; Yi and Abidian, 2015).

### Biomimicry strategy for efficient functional integration

**Figure 2:**
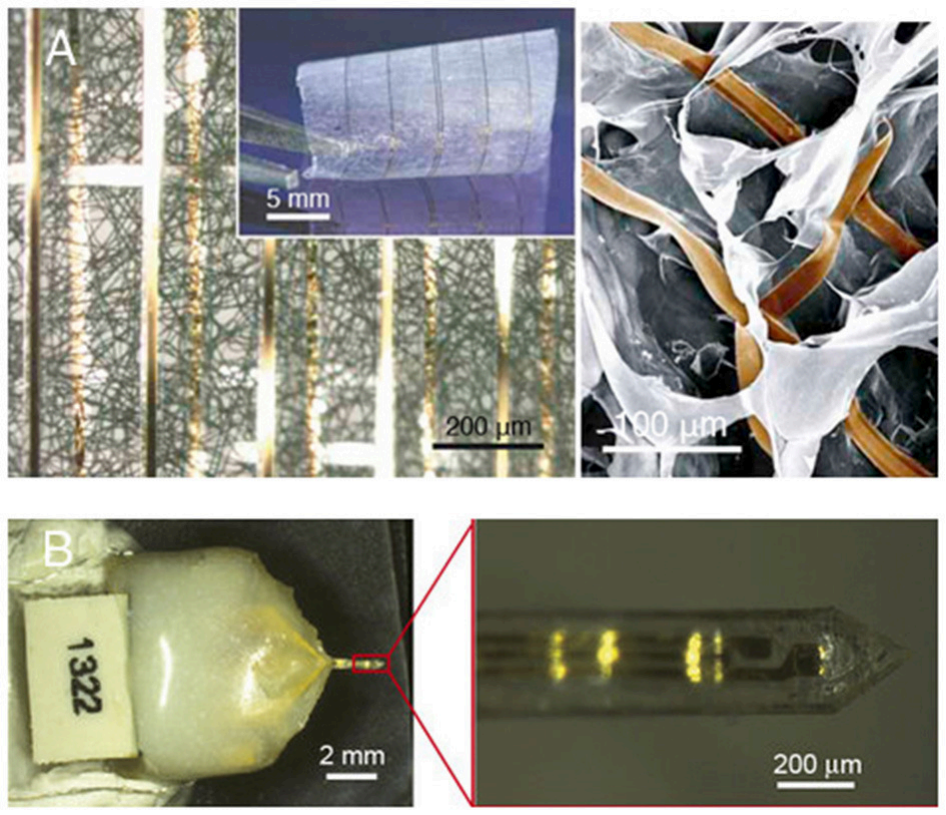
**Promising directions for neural interface design**. **(A)** Macroporous nanowire electrode array with integrated tissue scaffold (Tian et al., 2012), and **(B)** ECM-based microelectrodes (Shen et al., 2015). Copyright permissions were obtained from the respective publishers.

For efficient functional integration with a sufficient communication bandwidth, the requirements are (1) sufficient number of signal channels in the neural interface to resolve the full range of independent signal sources in the target neural tissue and (2) accessibility to each signal source by at least one neural electrode. For the first requirement, the constraints are the total percentage of tissue volumetric displacement by the high-density electrode probes and the unknown number of independent signal sources in the target neural tissue, so it is practical to have the highest channel count possible and drop off redundant informative during signal processing. In order to also comply to the insensibility principle, it is essential to employ nanoelectronics to achieve a high count of signal channels while minimizing the volume and area of materials used, as exemplified in a recent work (Figure 1B; Xie et al., 2015). The indistinguishability principle can be further applied by biochemical modification to the limited amount device materials. For the second requirement, the biomimicry engineering strategy is embodied in the design of regenerative neural interfaces for a more effective electrode–neuron integration through the creation of either tissue regeneration scaffolds in which micro neural electrodes are embedded (Figure 1D; Lacour et al., 2010; Clements et al., 2013; Musick et al., 2015; Srinivasan et al., 2015; Thompson et al., 2016) or muscle graft relays for guiding the deeply embedded neural signal sources to engineered structures for easily probing (French et al., 2014; Martin, 2015). These regenerative neural interfaces also manifest the need for dual-side engineering in order to create a better-integrated electrode-tissue interface, where tissue engineering and biomaterials can play a significant role in engineering the biologic side. In line with this, nanoelectronic scaffold neural interfaces hold great promise for both stable physical integration and efficient functional integration (Figure 2A; Tian et al., 2012).

## Future perspectives

Ultimately, to produce a long-term reliable neural interface with sufficient information bandwidth, it is necessary to integrate nanoelectronics with biomaterials and tissue engineering approaches. The use of abiologic materials should be minimized both in volume and area to comply with the insensibility principle. The inevitable abiologic materials should be camouflaged using biomimicry strategies to comply with the indistinguishability principle. Under such prerequisites, the high-bandwidth electronic functionalities should be implemented using nanoelectronics for hundreds or even thousands of signal channels (Zhang and Lieber, 2016). The resulting electrode-neural tissue interface essentially becomes a “cyber-tissue” (Figure 2A; Tian et al., 2012). An exciting recent work demonstrated an attempt toward this direction by constructing a three-dimensional macroporous nanoelectronic network with multiple embedded nanosensors, though the device materials have not been camouflaged biochemically (Figure 1B; Xie et al., 2015). Alternative, naturally derived biologic materials can be used to fabricate the neural interface (Chen and Allen, 2012), as recently demonstrated by fabricating intracortical microelectrodes using an ECM-based substrate material (Figure 2B; Shen et al., 2015), waiving the need to camouflage the abiologic material.

Then, one question comes up. What would the neural interface look like if we push the insensibility and indistinguishability principles to the extreme? In such a situation, there will be no abiologic and foreign materials, i.e., the device should be entirely made of autologous materials. As a result, there is no need to camouflage the device materials, as they are autologous and thus indistinguishable to the immune system and target neurons. Therefore, the technological challenges seem to be shifted to implementing the electronic functionalities using autologous materials, which, however, is infeasible in the foreseeable future. I would like to recall that there is no electronics in our body, yet our biologic body can perform sophisticated signal processing functionalities that are unachievable even with our most advanced integrated circuit systems. So, we won't need to build electronics; instead, we just need to implement the desired functionalities using autologous materials, i.e., to build biological circuits and living neural prostheses. Considering a convergence of synthetic biology, tissue engineering and neural prosthetics, such living neural prostheses will not be far from our reach in the foreseeable future.

## Author contributions

LG conceived the concept, analyzed the literature, wrote, and revised the manuscript.

### Conflict of interest statement

The author declares that the research was conducted in the absence of any commercial or financial relationships that could be construed as a potential conflict of interest.
